# Biological and Therapeutic Roles of Stem Cells in Head and Neck Carcinoma: Implications for Maxillofacial Surgery

**DOI:** 10.3390/diseases13120381

**Published:** 2025-11-22

**Authors:** Luca Michelutti, Alessandro Tel, Marco Zeppieri, Chiara Martinazzo, Massimo Robiony, Caterina Gagliano, Fabiana D’Esposito, Matteo Capobianco, Marieme Khouyyi

**Affiliations:** 1 Clinic of Maxillofacial Surgery, Head-Neck and NeuroScience Department, University Hospital of Udine, p.le S. Maria della Misericordia 15, 33100 Udine, Italy; micheluttiluca.uniud@gmail.com (L.M.);; 2 Department of Ophthalmology, University Hospital of Udine, 33100 Udine, Italy; 3 Department of Medicine, Surgery and Health Sciences, University of Trieste, 34127 Trieste, Italy; 4 Department of Medicine and Surgery, University of Enna “Kore”, Piazza dell’Università, 94100 Enna, Italy; 5 Eye Center “G.B. Morgagni-DSV”, 95125 Catania, Italy; 6 Imperial College Ophthalmic Research Group [ICORG] Unit, Imperial College, London NW1 5QH, UK; 7 Eye Clinic, Catania University, Policlinico G. Rodolico, Via Santa Sofia, 95121 Catania, Italy; 8 Faculty of Medicine, University of Catania, 95123 Catania, Italy; 9 Department of Biomedical and Dental Sciences and Morphofunctional Imaging, University of Messina, 98121 Messina, Italy

**Keywords:** cancer stem cells, head and neck carcinoma, maxillofacial surgery, molecular markers, precision medicine

## Abstract

**Background**: Head and neck carcinomas represent a heterogeneous group of aggressive malignancies with often poor prognosis and high recurrence rates. In recent years, the identification and characterization of cancer stem cells (CSCs) within these tumors have profoundly reshaped our understanding of tumorigenesis, resistance mechanisms, and metastatic potential in this anatomical district. Cancer stem cells (CSCs) play a central role in therapeutic resistance, recurrence, and metastatic progression in head and neck squamous cell carcinoma (HNSCC), particularly within the anatomically complex maxillofacial region. This review has synthesized recent advances in CSC biology, including marker heterogeneity, stemness-associated pathways, and interactions with the tumor microenvironment. **Methods**: A narrative review of the available literature was conducted, focusing on studies dealing with cancer stem cells in head and neck carcinoma and their implications for maxillofacial surgery. **Results**: We have critically examined emerging systemic and locoregional CSC-targeted therapies, highlighting inhibitors of Notch, Wnt/β-catenin, Hedgehog, and Hippo/YAP pathways, ALDH and ABC transporter inhibitors, autophagy modulators, nanoparticle-based delivery systems, and CSC-directed immunotherapies. The implications of these approaches for surgical planning, resection margins, and postoperative disease control in maxillofacial oncology have been discussed. To enhance clarity and analytical value, we have incorporated two comprehensive tables summarizing CSC markers and therapeutic strategies. Collectively, the evidence indicates that integrating CSC-oriented diagnostics and therapeutics into multimodal management may improve long-term outcomes for patients with maxillofacial HNSCC. **Conclusions**: This review highlights the critical need for integrating CSC-focused research into clinical practice to develop more effective, personalized, and durable treatment strategies. Such an approach could enhance oncologic control, reduce recurrence, and improve functional outcomes for patients undergoing complex oncologic procedures in the maxillofacial region.

## 1. Introduction

Head and neck squamous cell carcinomas (HNSCCs) account for approximately 900,000 new cases and over 400,000 deaths annually worldwide, making them the sixth most common cancer globally [1].

These malignancies arise from the mucosal epithelium of the oral cavity, oropharynx, hypopharynx, and larynx and are characterized by significant heterogeneity in etiology, molecular profile and clinical behavior [2]. Major risk factors include tobacco and alcohol consumption, as well as persistent infection with high-risk human papillomavirus (HPV), particularly HPV-16, which defines a distinct subset of oropharyngeal cancers with a generally more favorable prognosis [3].

Despite advances in surgery, radiotherapy, chemotherapy and, more recently, immunotherapy, the overall 5-year survival rate for HNSCC has remained at approximately 50–60% over the last three decades [4]. This stagnation is largely due to high rates of locoregional recurrence, distant metastases, and the development of resistance to standard therapies.

Increasing evidence indicates that a subpopulation of tumor cells known as cancer stem cells (CSCs) plays a pivotal role in these unfavorable clinical outcomes [5]. CSCs are defined by their ability to self-renew, differentiate into heterogeneous tumor cell populations and recapitulate the original tumor phenotype in vivo. They are thought to originate either from malignant transformation of normal tissue stem cells or from dedifferentiation of more differentiated tumor cells under selective pressures [6]. Crucially, CSCs exhibit intrinsic resistance to chemotherapy and radiotherapy through multiple mechanisms, including enhanced DNA damage repair, activation of anti-apoptotic pathways and expression of drug efflux pumps such as ATP-binding cassette (ABC) transporters [7].

In HNSCC, CSCs have been identified using a variety of surface markers (e.g., CD44, CD133) and functional assays (e.g., aldehyde dehydrogenase [ALDH] activity, sphere-forming ability). Their abundance has been correlated with poor clinical outcomes, increased risk of recurrence, and metastatic spread [8]. Moreover, CSCs contribute to epithelial–mesenchymal transition (EMT), a key process in invasion and metastasis, and engage in crosstalk with the tumor microenvironment to sustain an immunosuppressive niche [9].

For maxillofacial surgeons, the implications of CSC biology are profound. Understanding how CSCs influence tumor behavior could transform preoperative risk assessment, guide surgical margin determination, and inform the integration of CSC-targeted therapies into multimodal treatment plans. This review aims to provide a comprehensive and critical synthesis of current knowledge on CSCs in HNSCC, with a particular focus on their molecular characteristics, role in therapeutic resistance and translational implications for personalized maxillofacial oncologic surgery.

This review focuses on stemness features and biomarkers in head and neck malignancies that directly influence resistance, prognosis, and treatment tailoring, rather than presenting a comprehensive list of all CSC indicators. We specifically highlight how CSC-related molecular characteristics might be utilized to enhance risk classification, customize multimodal treatment, and guide surgical planning in patients with head and neck squamous cell carcinoma.

## 2. Biology of Cancer Stem Cells in HNSCC

Cancer stem cells (CSCs) are a subpopulation of tumor cells with the ability to self-renew, sustain tumor growth and recreate tumor heterogeneity [10]. Their role in malignancy has been investigated in vitro, in vivo, and in clinical studies. In particular, the expression of specific CSC markers and stemness-associated genes has been correlated with histological grade, tumor stage, and the presence of metastasis in HNSCC patients. Experimental models to study CSC function are diverse and include two-dimensional human and animal cell lines/strains, three-dimensional organoids, and xenograft models in immunodeficient mice [11].

The principal markers identified for HNCSCs are CD44, Aldehyde Dehydrogenase 1 (ALDH1), and CD133 [12]. Additional potential CSC surface markers later identified include CD10, CD98, CD271, CD166 and ABCG2. HNCSCs also express numerous genes typical of normal stem cells, such as embryonic stem cell transcription factors SOX2, OCT4 and NANOG, supporting their multipotency and self-renewal capabilities (Table 1) [10].

Over the past decade, high-resolution technologies, including single-cell RNA sequencing, lineage-tracing models, and spatial transcriptomics, have enhanced the traditional understanding of cancer stem cells in head and neck squamous cell carcinoma. These methodologies have shown that stem-like characteristics can be dynamically gained and relinquished over a spectrum of cellular states, rather than being confined to a static small subpopulation. Supplementary potential markers, such as CD24, CD47, CXCR4, and the oncofetal antigen 5T4, have been associated with self-renewal, immune evasion, and metastatic dissemination in head and neck malignancies. Simultaneously, functional signatures obtained from stemness-associated transcriptional programs have demonstrated superior efficacy compared to individual markers in forecasting recurrence and treatment failure. These observations endorse a more cohesive understanding of cancer stem cells (CSCs), wherein marker expression, epigenetic adaptability, and microenvironmental signals converge to influence tumor behavior, yielding significant implications for prognosis, therapeutic response, and the formulation of CSC-targeted strategies in the maxillofacial context.

CSCs maintain stemness and prevent cell differentiation through epigenetic mechanisms. These include the overexpression of EZH2, a histone methyltransferase that function as a subunit of the Polycomb Group (PcG) proteins—a family of transcriptional repressors involved in the regulation of gene expression. EZH2 overexpression is associated with aggressiveness and less favorable prognosis in oral cancer. Similarly, BMI1 (another Polycomb Group protein) promotes chromatin silencing. Epigenetic mechanisms are also involved in the deregulation of the Wnt/β-catenin pathway and the upregulation of several genes associated with Notch and Hedgehog [13].

A key mechanism by which CSCs promote tumor invasion and metastasis is through the epithelial-to-mesenchymal transition (EMT), during which HNSCC cells lose epithelial features and acquire a more invasive mesenchymal phenotype. CSCs gain enhanced motility and the ability to breach the basement membrane, facilitating tumor dissemination and metastatic spread [10]. Studies have identified different CSC subpopulations, with one subgroup showing a migratory EMT-like phenotype by high expression of EMT-related genes and proteins (Vimentin, Snail, Twist) and another subgroup presenting an epithelial-like, highly proliferative phenotype. These subgroups can switch between EMT and MET, which promotes metastatic dissemination and tumor growth in secondary sites [11]. Analogous to normal stem cells, cancer stem cells are also believed to reside in specialized microenvironments, known as CSC niches, which support their maintenance, survival and functional properties [14].

The CSC niche is often located near blood vessels and includes both cellular and non-cellular components, each crucial for maintaining CSCs properties. Key cellular components include endothelial cells, which secrete growth factors that stimulate the expression of stemness-associated genes; immune cells such as T cells, tumor-associated macrophages (TAM) and myeloid-derived suppressor cells (MDSC); and fibroblasts, which can be transformed into cancer-associated fibroblasts (CAFs) that secrete MMP-2 and MMP-9, metalloproteinases involved in EMT, stemness and metastasis [11,14,15]. Non cellular components include growth factors, matrix metalloproteinases, various cytokines and chemokines such as CXCL2, which recruit inflammatory cells. Vascular endothelial growth factor, produced by CAFs and endothelial cells, plays a crucial role in promoting angiogenesis [10]. Another growth factor, EGF, has been shown to promote stemness, induce EMT, and stimulate the expression of several CSC-related factors, such as BMI1, ALDH [11]. CSCs themselves secrete cytokines, including TGFb-1 and IL-6, which modulate the tumor microenvironment with their anti-inflammatory properties.

The tumor microenvironment (TME) is crucial in maintaining cancer stem cell (CSC) phenotypes in head and neck squamous cell carcinoma (HNSCC). Bidirectional communication between cancer stem cells (CSCs) and cancer-associated fibroblasts (CAFs) amplifies stemness via paracrine stimulation of the IL-6/STAT3, TGF-β/SMAD, and CXCL12/CXCR4 signaling pathways. Tumor-associated macrophages (TAMs), especially the M2-polarized variant, enhance cancer stem cell (CSC) survival by secreting EGF, VEGF, and CCL2, while also facilitating immunosuppression through the activation of PD-L1. Hypoxia, a characteristic of the maxillofacial tumor microenvironment, enhances the stabilization of HIF-1α and HIF-2α, promoting epithelial–mesenchymal transition (EMT), metabolic reprogramming, and resistance to chemoradiotherapy. Perivascular habitats serve as repositories for cancer stem cells, with endothelial cells facilitating tumor-initiating potential via NOTCH ligand-mediated interactions.

Therapeutically, interrupting CSC–TME signaling has surfaced as a viable approach. Inhibitors of IL-6/STAT3, CXCR4 antagonists, and TGF-β blockade have shown preclinical effectiveness in decreasing CSC frequency, modifying niche composition, and reinstating susceptibility to cytotoxic therapy. Targeting hypoxia with HIF inhibitors or hypoxia-activated prodrugs has demonstrated ability to disrupt stemness programs. TAM-modulating drugs, such as CSF1R inhibitors and CD40 agonists, have transformed the immune milieu to promote anti-tumor action. Anti-angiogenic drugs that restore tumor vasculature have also diminished CSC-endothelial signaling. These approaches collectively endorse a multi-faceted therapeutic framework that integrates CSC-targeted tactics with TME-focused therapies to disrupt the reciprocal reinforcement contributing to treatment failure.

In addition, CSCs-derived exosomes (CSCEX) display immunosuppressive properties, although their role in escaping immunosurveillance remains to be fully clarified [16].

Furthermore, recent studies focusing on oral microbiota suggest that specific oral microbiota, including HPV, *Porphyromonas gingivalis* (PG), *Fusobacterium nucleatum* (FN), and other yet unidentified species, may promote stemness in oral cancer cells and modulate CSC niche defense by activating tumor stemness pathways and altering epigenetic mechanisms [17].

An additional factor enhancing CSC survival is hypoxia, which stimulates the expression of transcription factors such as HIF1α that increase VEGF production and reinforce stemness, EMT, and niche interaction [10].

A range of experimental instruments has been created to identify and functionally characterize cancer stem cells in head and neck squamous cell carcinoma. Sphere-formation tests and clonogenic assays are the predominant methodologies employed to evaluate self-renewal and tumor-initiating potential. The ALDEFLUOR assay facilitates the functional quantification of ALDH high cancer stem cell populations, whilst flow cytometry-based sorting employing markers like CD44, ALDH1, and CD133 permits the isolation and subsequent study of CSC-enriched fractions. Advanced methods such as lineage-tracing models, single-cell RNA sequencing, and spatial transcriptomics have yielded profound insights into cancer stem cell plasticity, intratumoral heterogeneity, and microenvironmental interactions. These strategies have significantly enhanced our comprehension of CSC behavior and are crucial for validating targets of CSC-directed therapy.

This review does not intend to summarize every phenotypic trait documented in the literature, but instead to emphasize the CSC-associated molecules and pathways that are most pertinent for therapeutic application. By concentrating on indicators associated with therapeutic resistance, locoregional recurrence, and dissemination patterns that pose significant challenges in the maxillofacial region, we aim to establish a biologically informed framework to aid clinicians in interpreting CSC data and incorporating it into routine decision-making.

Despite numerous studies proposing CSC markers in HNSCC, the evidence remains heterogeneous, exhibiting significant variability in methodologies, sampling protocols, and functional validation. Numerous markers are from limited cohorts or in vitro models that may inadequately represent the geographical and temporal flexibility of cancer stem cells in vivo. These constraints must be considered when converting CSC signals into predictive or therapeutic instruments in the craniofacial context.

## 3. Therapeutic Strategies, Resistance and Future Therapies

### 3.1. Systemic Therapeutic Strategies Targeting Cancer Stem Cells in HNSCC

Treatment of head and neck squamous cell carcinoma (HNSCC) depends on disease stage. Early-stage HNSCC is mainly treated with surgery, sometimes combined with radiotherapy or cisplatin-based chemotherapy in high-risk cases. Advanced disease requires multimodal approaches, with cisplatin remaining the standard backbone, often paired with 5-FU, taxanes, or cetuximab. Although numerous CSC-targeted systemic therapies have shown encouraging preclinical efficacy, the majority are still substantiated by preliminary studies with restricted clinical validation. The variability in dose, delivery, and CSC measurement methodologies among research hinders direct comparison, highlighting the necessity for stringent, standardized trials that explicitly assess CSC depletion, recurrence patterns, and functional surgical outcomes.

In recurrent or metastatic settings, systemic therapy is the only option but yields modest survival benefits and high toxicity. Immune checkpoint inhibitors (nivolumab, pembrolizumab) provide durable responses in a minority of patients, while targeted therapies such as tipifarnib, tyrosine kinase inhibitors, and CDK4/6 inhibitors are still investigational [18].

Cancer stem cells (CSCs) in HNSCC display enhanced resistance to chemotherapy, largely due to their intrinsic stemness properties. These cells are characterized by high expression of stemness-associated markers, including SOX2, Bmi-1, CD44, Nanog, Oct4, and aldehyde dehydrogenase (ALDH), which contribute to the survival of CSCs during cisplatin treatment and other chemotherapeutic regimens [11,18,19]. Autophagy has also been implicated as a critical process for maintaining CSC stemness and promoting chemoresistance [19].

The epithelial-to-mesenchymal transition (EMT) further supports drug resistance. EMT-related transcription factors, such as SOX8, enhance both stemness and cisplatin resistance, while signaling pathways like Hedgehog and Hippo/YAP-TAZ contribute to the maintenance and enrichment of CSC-like populations [18,19]. Key regulators of drug efflux, including ABC transporters (ABCB1, ABCB11, MDR1, MRP-1, ABCG2), are frequently upregulated in CSCs, facilitating the extrusion of chemotherapeutic agents and reinforcing resistance [10,11,19]. Exosomal miRNAs, such as miR-155, also modulate drug efflux and stemness-associated pathways [19].

Metabolic reprogramming represents another mechanism of chemoresistance. CSCs often rely on enhanced glycolysis and antioxidant defenses, mediated by pathways such as YAP1/TEAD1/GLUT1 and c-Myc/PLK1/Akt, which sustain proliferation and reduce susceptibility to chemotherapeutic-induced oxidative stress [10,18,19]. Low levels of reactive oxygen species (ROS) in CSCs further protect against DNA damage induced by cisplatin [10].

Additional contributors to chemoresistance include increased expression of anti-apoptotic proteins, such as cIAP-1, cIAP-2, and XIAP, and enhanced DNA repair activity through checkpoint kinases CHEK1 and CHEK2, which allow CSCs to survive chemotherapy-induced genotoxic stress [10,11]. Inflammatory signals, particularly IL-6 derived from endothelial cells or induced by cisplatin itself, can expand the pool of BMI1-positive CSCs, further sustaining resistance [18].

Altogether, these features—stemness, EMT, drug efflux, metabolic adaptation, enhanced DNA repair, and anti-apoptotic signaling—converge to make CSCs highly resilient to chemotherapy. Targeting these pathways, including ALDH1, NRF2, and TGFβ signaling, holds promise for overcoming chemoresistance and improving therapeutic outcomes in HNSCC [18].

Cancer stem cells (CSCs) in HNSCC show marked resistance to radiotherapy, which contributes to tumor persistence and relapse. Recent studies have highlighted the role of key stemness regulators such as SALL4. In CD44-positive OSCC cells, SALL4 promotes nuclear translocation of β-catenin and activates downstream Wnt/β-catenin target genes, thereby reinforcing the CSC phenotype and increasing resistance to radiation [20]. METTL3 has been shown to upregulate SALL4 expression, establishing a METTL3/SALL4/Wnt/β-catenin axis that supports CSC maintenance and radioresistance, ultimately facilitating OSCC progression [20].

Beyond specific transcriptional programs, CSCs exhibit slow-cycling behavior, enhanced DNA repair through checkpoint activation, and increased anti-apoptotic signaling, all of which contribute to their survival under radiation-induced genotoxic stress [21]. Moreover, radiation may induce non-CSCs to acquire CSC-like properties, indicating a dynamic plasticity that further complicates therapeutic eradication [21].

These findings underscore that both molecular pathways and functional traits of CSCs converge to confer radioresistance in HNSCC, emphasizing the need for therapies that specifically target CSC populations to improve radiotherapy outcomes.

Recent advances in understanding cancer stem cell (CSC) biology in HNSCC have highlighted multiple potential therapeutic strategies aimed at overcoming chemoresistance and radioresistance. Several promising molecular targets are currently under investigation. Inhibitors of the TGFβ and NRF2 pathways, as well as molecules targeting ALDH1, have shown potential in preclinical and early clinical studies to reduce CSC stemness and enhance sensitivity to cisplatin-based chemotherapy [19]. Autophagy, which modulates CSC stemness and interacts with ROS-regulated signaling pathways such as mTOR, JNK, STAT3, and AKT, is also being explored as a therapeutic target. Nanoparticle-based drug delivery systems offer additional opportunities for selective targeting of CSCs, for instance via integrin α5 or β-catenin, providing tumor-specific delivery and potentially overcoming cisplatin resistance [19].

Immunotherapeutic approaches represent another promising avenue. Vaccines based on ITGB4-expressing dendritic cells (mITGB4-DC) or adoptive transfer of BiAb-armed T cells targeting ITGB4 have been shown to suppress both CSCs and bulk tumor cells in preclinical models, reducing local tumor growth and metastasis [22].

Checkpoint modulation and restoration of tumor suppressor pathways also offer potential strategies. MDM2 inhibitors can reactivate endogenous wild-type p53, promoting CSC differentiation, decreasing the CSC population, and enhancing response to chemotherapy [23]. Targeting the Wnt/β-catenin, Hedgehog, and Hippo/YAP pathways, previously implicated in chemoresistance and radioresistance, may further prevent CSC survival and expansion [18,19,20].

The primary categories of CSC-directed methods now being examined in HNSCC and their mechanisms of action are delineated in Table 2 to aid in their application in maxillofacial oncologic practice.

Overall, these approaches—spanning molecular inhibition, autophagy modulation, nanoparticle-mediated drug delivery, immunotherapy, and tumor suppressor reactivation—aim to selectively eradicate CSC populations or reduce their stemness. Combining these CSC-targeted strategies with conventional chemotherapy or radiotherapy holds promise for overcoming resistance, minimizing relapse, and improving patient outcomes in HNSCC.

Insights gained from previous cancers have elucidated the prospects and constraints of CSC-targeted treatments. In acute myeloid leukemia (AML), the targeting of the FLT3 and BCL-2 pathways has indirectly diminished leukemic stem cells, leading to significant enhancements in survival with medicines like venetoclax. In breast cancer, the suppression of HER2 and PI3K/AKT signaling has diminished cancer stem cell activity and facilitated sustained responses in specific molecular subtypes. In contrast, numerous tactics have been shown to be less effective. In glioblastoma, Hedgehog and Notch pathway inhibitors have demonstrated minimal clinical efficacy despite strong preclinical outcomes, possibly attributable to significant cancer stem cell plasticity and microenvironmental protection. Clinical studies for pancreatic cancer targeting CD44 or CXCR4 have encountered toxicity, suboptimal target engagement, or inadequate penetration into thick stromal environments. These triumphs and failures demonstrate that effective CSC regulation necessitates the concurrent targeting of stemness pathways, microenvironmental support, and immune evasion mechanisms. These insights are particularly pertinent to head and neck tumors in the maxillofacial region, where analogous patterns of plasticity, stromal interaction, and immune evasion hinder therapeutic application.

### 3.2. Locoregional Pharmacological Administration and Cancer Stem Cell Targeting in Maxillofacial Oncology

Alongside systemic therapy, head and neck squamous cell carcinomas are amenable to locoregional drug delivery methods that utilize the anatomical accessibility of the upper aerodigestive tract. Superselective intra-arterial infusion, intratumoral injection, topical administration of chemotherapeutic drugs, and drug-eluting implants have been investigated to enhance local medication concentrations while minimizing systemic toxicity. These modalities are especially appealing in the context of CSC-targeted therapies, as CSCs frequently inhabit perivascular niches and the invasive tumor front, where locally administered medicines might be concentrated.

Superselective intra-arterial chemotherapy enables the direct high-dose infusion of cisplatin and other drugs into the arterial supply of oral cavity and oropharyngeal cancers, resulting in significant tumor reduction in certain studies and enhancing organ preservation rates. Integrating intra-arterial administration with drugs that obstruct CSC-related pathways or efflux pumps may improve the eradication of CSCs at the invasive front, thereby diminishing the probability of positive margins and locoregional recurrence post-resection. For maxillofacial surgeons, such protocols may result in more advantageous tumor biology during surgery and enable function-preserving operations.

The intratumoral and topical delivery of chemotherapeutic or biological drugs has been explored, such as through direct injection into exophytic or ulcerated oral lesions, or by putting drug-laden gels and films on the mucosal surface. These methodologies may be especially effective for administering nanoparticles, siRNA constructs, or small-molecule inhibitors that selectively target cancer stem cells and their microenvironments, including the oral microbiota and related inflammatory conditions. The treated area is readily observable and accessible, allowing for close monitoring of response and adjustment of dose based on local tolerability and tumor reduction.

An additional potential approach in maxillofacial cancer is the implementation of drug-eluting scaffolds, plates, or meshes inserted into the resection cavity post-tumor excision. Biodegradable carriers containing cisplatin, pathway inhibitors, or CSC-targeted immunotherapeutic medicines may deliver persistent, elevated local drug concentrations at areas where residual CSCs are anticipated to remain, hence reducing systemic exposure. These implants can be used in reconstructive surgeries (e.g., free flap reconstruction, bone grafting) and may provide a method to combine structural restoration with biological management of residual disease.

Despite these benefits, the clinical application of locoregional CSC-targeted treatments is still in its nascent phase. The variability in catheterization techniques, dose regimens, and carrier materials, along with a scarcity of randomized data, now obstructs the standardization of these methods. Moreover, effective biomarkers that might identify patients likely to gain the most advantage from CSC-focused locoregional treatment are yet absent. Subsequent research should emphasize the formulation of trials that use CSC profiling, sophisticated imaging, and locoregional delivery techniques, with particular focus on functional and aesthetic results in the craniofacial region. Maxillofacial surgeons must collaborate closely with medical and radiation oncologists to properly schedule and customize these procedures within multimodal therapy strategies.

Notwithstanding promising preclinical advancements, considerable obstacles hinder the integration of CSC-targeted therapies into clinical practice. The variability and adaptability of CSC indicators hinder the creation of a universally applicable, clinically proven diagnostic signature. Many existing indicators exhibit poor repeatability across cohorts and frequently vary with therapy, hypoxia, and microenvironmental influences. Secondly, the availability of effective and scalable clinical assays for assessing cancer stem cell burden before and after therapy is limited, hence complicating patient selection and treatment monitoring. Third, CSC-targeted pharmaceuticals often interact with developmental pathways that may exhibit toxicities in normal stem cell compartments, prompting safety concerns that necessitate meticulously constructed dose-finding experiments. Furthermore, the lack of validated endpoints—such as CSC depletion, delayed recurrence from CSC clones, or biomarker-driven stratification—has impeded the progression of clinical trials. Ultimately, the incorporation of CSC-directed medicines into multimodal head and neck cancer treatment necessitates synchronization with surgical procedures, reconstructive planning, and radiotherapy timetables, thereby increasing logistical complexity. Surmounting these obstacles necessitates standardized tests, real-time CSC monitoring, strategic combination regimens, and biomarker-integrated trial designs.

## 4. Clinical Implications of CSC in HNSCC: Toward Personalized Maxillofacial Surgery

The clinical relevance of cancer stem cells (CSCs) in head and neck squamous cell carcinoma (HNSCC) is increasingly evident. High CSC content has been associated with tumor aggressiveness, therapy resistance, and unfavorable survival outcomes [5,8]. For example, patients with tumors enriched in CD44 high/ALDH1+ subpopulations experience increased recurrence and metastasis, even after standard multimodal therapy [24].

A large meta-analysis confirmed that overexpression of CSC markers such as Bmi-1, CD133, Nanog and Oct-4 significantly correlates with poor overall survival and disease-free survival in HNSCC patients [25]. These findings highlight that the TNM system alone cannot fully predict outcomes, reinforcing the need to integrate CSC profiling into clinical practice.

### 4.1. CSC Profiling for Surgical Planning

In maxillofacial oncology, positive margins remain one of the strongest predictors of recurrence. CSCs are frequently concentrated at the invasive tumor front and within perivascular niches, areas that may escape conventional imaging and histopathological evaluation [24].

CSC profiling by immunohistochemistry (CD44, SOX2, OCT4, NANOG), functional assays (ALDH1 activity), or transcriptomic signatures could complement preoperative workups. Emerging technologies, including intraoperative molecular diagnostics and point-of-care assays, may allow real-time detection of CSC markers during surgery, guiding margin control and reducing re-excision rates [26]. This integration of biological information into surgical planning could foster a precision surgery paradigm, balancing oncologic safety with functional preservation.

### 4.2. Neoadjuvant and Adjuvant CSC-Targeted Approaches

In the neoadjuvant setting, preclinical studies show that targeting CSC-related pathways such as Notch, Wnt/β-catenin and Hedgehog can reduce CSC frequency and sensitize tumors to chemotherapy and radiotherapy [27]. Such strategies could facilitate more radical resections and lower recurrence risk.

In the adjuvant setting, therapies designed to eradicate residual CSCs could complement standard chemoradiation. Examples include monoclonal antibodies against CD44, ALDH inhibitors, and nanoparticle-based delivery systems, which have shown efficacy in selectively eliminating CSC subpopulations [28].

Recent work has also explored novel immunotherapeutic strategies, including approaches targeting the oncofetal antigen 5T4, a glycoprotein enriched in cancer stem cell populations. In preclinical HNSCC models, 5T4-targeted therapy was shown to ablate CSCs and prevent tumor recurrence [29], and recent systematic reviews have identified 5T4 as a promising CSC regulator and therapeutic target in head and neck cancer an [30].

Together, these findings support a multimodal treatment paradigm, in which CSC-targeted strategies are integrated with surgery to improve long-term control.

### 4.3. Long-Term Stratification and Personalized Follow-Up

CSCs contribute to late recurrences, even years after apparently successful therapy. Consequently, personalized follow-up strategies are needed: liquid biopsies (circulating tumor cells, ctDNA, extracellular vesicles) are being evaluated as minimally invasive tools for detecting minimal residual disease enriched in CSC signatures [31], dynamic stratification models that combine CSC biomarkers with radiological and clinical data can refine recurrence risk and guide individualized follow-up [24], and AI-based approaches integrating CSC molecular profiles with imaging (radiomics) are emerging as future tools for predicting relapse and optimizing surveillance [2].

Patients with persistent CSC signals may benefit from intensified surveillance and adjuvant interventions, while those with low CSC burden could follow standard protocols, reducing overtreatment. Such an approach could shift maxillofacial oncology from an anatomically driven model toward a biologically guided precision medicine framework, improving both oncologic control and functional outcomes.

## 5. Current Limitations and Future Perspectives

Although research has made significant progress in recent years in clarifying the role of cancer stem cells (CSCs) in head and neck carcinomas, the clinical application of this knowledge still faces many limitations and obstacles.

A first critical issue concerns the identification of CSCs themselves. The proposed markers—such as CD44, CD133, ALDH1, and others—are neither unique nor exclusive, and their expression varies considerably. This heterogeneity, combined with cellular plasticity that allows neoplastic cells to acquire and lose stem-like characteristics in response to the environment or treatments, makes it difficult to standardize reliable and universally applicable diagnostic tests on a large scale.

A further limitation stems from the experimental models currently available. Much of the evidence comes from two-dimensional cell cultures, organoids, or murine xenografts, which, while providing valuable information, do not fully reflect the biological and microenvironmental complexity of human tumors. The absence of validated and standardized preclinical models represents a real obstacle to the translation of anti-CSC strategies into everyday clinical practice.

The integration of CSC profiling into surgical planning for cancer patients is also still at an experimental stage. Although immunohistochemical tests and functional assays (e.g., ALDH activity) have shown incredible potential, there is a lack of rapid and reliable intraoperative tools capable of guiding decisions on resection margins in real time. Similarly, the clinical utility of incorporating CSC assessment into personalized follow-up protocols requires large-scale prospective validation.

The future outlook, however, appears promising. On the one hand, liquid biopsy techniques—which allow the detection of circulating tumor cells, cell-free tumor DNA, or extracellular vesicles enriched in molecular signatures of CSCs—could enable noninvasive and dynamic monitoring of residual disease and risk of recurrence. On the other hand, advances in nanotechnology are paving the way for targeted drug delivery systems capable of selectively targeting CSCs while sparing healthy tissue. At the same time, immunotherapeutic approaches—such as cellular vaccines directed against CSC-associated antigens or the use of T cells armed with bispecific antibodies—are showing encouraging results in preclinical models.

Artificial intelligence will also play a crucial role. The integration of molecular data, radiomic imaging, and clinical parameters using predictive algorithms could refine patient stratification, personalize treatment choices, and optimize post-operative surveillance protocols. For these prospects to become reality, it will be essential to promote interdisciplinary collaborations, implement standardized protocols, and develop multicenter clinical trials capable of validating the efficacy and safety of anti-CSC approaches in real-world settings.

## 6. Conclusions

Squamous cell carcinoma of the head and neck remains one of the most complex and insidious neoplasms, characterized by high biological aggressiveness, frequent recurrence, and long-term survival that is not always satisfactory. In this scenario, cancer stem cells play a central role in neoplastic progression, resistance to conventional treatments, and metastatic spread.

Their characteristics—capacity for self-renewal, epithelial–mesenchymal plasticity, metabolic remodeling, and interaction with an immunosuppressive microenvironment—make them both a fascinating therapeutic target and an unresolved challenge. For maxillofacial surgeons, understanding these mechanisms not only is of theoretical value but also has potential practical implications for planning surgery, intraoperative margin management, and, above all, personalizing postoperative follow-up.

While difficulties related to marker standardization and clinical validation are slowing widespread application, the horizon of therapeutic innovations is rapidly expanding. Molecules capable of inhibiting stemness-related signaling pathways, nanoparticle-based drug delivery systems, immunotherapies targeting CSC-specific antigens, and predictive models based on artificial intelligence point to a future in which head and neck cancer surgery will be guided not only by anatomical criteria, but also by biological and molecular parameters.

Future investigations into CSC-targeted strategies in HNSCC must emphasize the creation of cohesive diagnostic platforms that amalgamate molecular profiling, spatial transcriptomics, and real-time CSC quantification. These technologies would facilitate patient categorization, treatment surveillance, and adaptive therapy. Secondly, combination techniques that concurrently target stemness pathways, metabolic reprogramming, and survival signals emanating from the tumor microenvironment constitute a logical progression for enhancing cancer stem cell eradication. Third, locoregional delivery systems—such as drug-eluting scaffolds, nanoparticles, and intra-arterial infusion protocols—must be carefully assessed to ascertain if localized intensification can mitigate recurrence risk in anatomically intricate craniofacial regions. Clinical trial designs must integrate CSC-specific objectives, such as alterations in CSC load, postponed recurrence stemming from CSC-rich niches, and associations with surgical margin biology. Collaborative, multi-institutional research will be crucial to address the heterogeneity of HNSCC and to validate biomarkers across various surgical and treatment settings. Ultimately, the integration of CSC-directed therapies with precision surgery, immunotherapy, and microenvironmental modification may yield more permanent, function-preserving outcomes for patients with craniofacial malignancies.

The integration of knowledge about CSCs into a precision medicine paradigm could result in a twofold benefit: improving long-term cancer control and maximizing the function and quality of life of patients. The challenge for the coming years will be to transform these promising scientific insights into concrete tools for everyday clinical practice.

## Figures and Tables

**Table 1:** Principal CSC markers in HNSCC and clinical relevance for maxillofacial oncology.

Marker	Main Biological Role in HNSCC	Representative Evidence	Implications for Maxillofacial Surgery
CD44	Cell adhesion, migration, maintenance of stemness; enriched in tumor-initiating cells	Associated with poor differentiation, higher T stage, and increased locoregional recurrence	May indicate higher risk of positive/close margins at the invasive front; supports wider resections and intensified surveillance
ALDH1	Detoxification of aldehydes, ROS regulation, stemness maintenance	Correlates with radio- and chemoresistance, early recurrence, and decreased survival	Suggests the presence of treatment-resistant clones; relevant for selecting patients for intensified adjuvant therapy
CD133	Tumor initiation and angiogenesis; enrichment in metastatic subclones	Linked to nodal metastasis and distant spread in several HNSCC series	May guide nodal dissection strategies and indicate higher need for multimodal treatment
SOX2, OCT4, NANOG	Core stemness transcription factors, EMT promotion	Overexpression associated with aggressive behavior, recurrence, and reduced overall survival	Could be incorporated into prognostic panels to refine risk stratification and follow-up intensity
5T4, ITGB4	Oncofetal antigen and integrin implicated in CSC phenotype and immune evasion	Identified as CSC-associated targets in preclinical HNSCC models; amenable to immunotherapy	Provide rational targets for CSC-directed systemic or locoregional therapies in conjunction with surgery

CD: Cluster of differentiation; CSC: Cancer stem cell; HNSCC: Head and neck squamous cell carcinoma; ALDH: Aldehyde dehydrogenase; SOX2: SRY-box transcription factor 2; OCT4: Octamer-binding transcription factor 4; NANOG: Nanog homeobox; EMT: Epithelial–mesenchymal transition; ITGB4: Integrin beta-4; 5T4: Oncofetal trophoblast glycoprotein; ROS: Reactive oxygen species.

**Table 2:** Emerging CSC-targeted therapeutic strategies in HNSCC and potential relevance for maxillofacial surgery.

Strategy/Agent Class	Primary CSC-Related Target/Pathway	Mechanism of CSC Inhibition	Potential Clinical Application in Maxillofacial Oncology
Notch, Wnt/β-catenin, Hedgehog, Hippo/YAP inhibitors	Developmental signaling pathways sustaining stemness	Reduce self-renewal, sphere formation, and CSC frequency; sensitize to cisplatin/radiation	Neoadjuvant or adjuvant use to debulk CSCs and improve durability of surgical control
ALDH and ABC transporter inhibitors	ALDH1, ABCG2, MDR1	Impair detoxification and drug efflux; increase intracellular drug accumulation and apoptosis	Combination with perioperative chemotherapy to target residual CSCs in surgical margins
Autophagy modulators	Autophagy machinery (e.g., mTOR-regulated pathways)	Disrupt pro-survival autophagy in CSCs; enhance DNA damage and cell death	Adjunct to chemoradiotherapy to prevent CSC-mediated recurrence after resection
Nanoparticle-based drug delivery	Integrins, CD44, β-catenin-related components	Targeted delivery of cytotoxic or pathway inhibitors to CSC niches	Locoregional administration into the tumor bed or resection cavity to concentrate CSC-directed agents
CSC-directed immunotherapies (vaccines, BiAb-armed T cells)	CSC-associated antigens (e.g., ITGB4, 5T4)	Immune-mediated recognition and killing of CSCs	Perioperative integration to eradicate minimal residual disease and reduce late relapse

CSC: Cancer stem cell; HNSCC: Head and neck squamous cell carcinoma; ALDH: Aldehyde dehydrogenase; ABC: ATP-binding cassette; ABCG2: ATP-binding cassette subfamily G member 2; MDR1: Multidrug resistance protein 1; mTOR: Mechanistic target of rapamycin; BiAb: Bispecific antibody.

## Data Availability

The data are available upon request.

## References

[1] Sung H., Ferlay J., Siegel R.L., Laversanne M., Soerjomataram I., Jemal A., Bray F. (2021). Global Cancer Statistics 2020: GLOBOCAN Estimates of Incidence and Mortality Worldwide for 36 Cancers in 185 Countries. CA Cancer J. Clin..

[2] Leemans C.R., Snijders P.J.F., Brakenhoff R.H. (2018). The Molecular Landscape of Head and Neck Cancer. Nat. Rev. Cancer.

[3] Gillison M.L., Chaturvedi A.K., Anderson W.F., Fakhry C. (2015). Epidemiology of Human Papillomavirus–Positive Head and Neck Squamous Cell Carcinoma. J. Clin. Oncol..

[4] Cramer J.D., Burtness B., Le Q.T., Ferris R.L. (2019). The Changing Therapeutic Landscape of Head and Neck Cancer. Nat. Rev. Clin. Oncol..

[5] Prince M.E., Sivanandan R., Kaczorowski A., Wolf G.T., Kaplan M.J., Dalerba P., Weissman I.L., Clarke M.F., Ailles L.E. (2007). Identification of a Subpopulation of Cells with Cancer Stem Cell Properties in Head and Neck Squamous Cell Carcinoma. Proc. Natl. Acad. Sci. USA.

[6] Zhou H.-M., Zhang J.-G., Zhang X., Li Q. (2021). Targeting Cancer Stem Cells for Reversing Therapy Resistance: Mechanism, Signaling, and Prospective Agents. Signal Transduct. Target. Ther..

[7] Baumann M., Krause M., Hill R. (2008). Exploring the Role of Cancer Stem Cells in Radioresistance. Nat. Rev. Cancer.

[8] Chen Y.C., Chen Y.W., Hsu H.S., Tseng L.M., Huang P.I., Lu K.H., Chen D.T., Tai L.K., Yung M.C., Chang S.C. (2009). Aldehyde Dehydrogenase 1 Is a Putative Marker for Cancer Stem Cells in Head and Neck Squamous Cancer. Biochem. Biophys. Res. Commun..

[9] Mani S.A., Guo W., Liao M.-J., Eaton E.N., Ayyanan A., Zhou A.Y., Brooks M., Reinhard F., Zhang C.C., Shipitsin M. (2008). The Epithelial-Mesenchymal Transition Generates Cells with Properties of Stem Cells. Cell.

[10] Birkeland A.C., Owen J.H., Prince M.E. (2015). Targeting Head and Neck Cancer Stem Cells: Current Advances and Future Challenges. J. Dent. Res..

[11] Cirillo N., Wu C., Prime S.S. (2021). Heterogeneity of Cancer Stem Cells in Tumorigenesis, Metastasis, and Resistance to Antineoplastic Treatment of Head and Neck Tumours. Cells.

[12] Yu S.S., Cirillo N. (2020). The Molecular Markers of Cancer Stem Cells in Head and Neck Tumors. J. Cell Physiol..

[13] Castilho R.M., Squarize C.H., Almeida L.O. (2017). Epigenetic Modifications and Head and Neck Cancer: Implications for Tumor Progression and Resistance to Therapy. Int. J. Mol. Sci..

[14] Oshimori N. (2020). Cancer Stem Cells and Their Niche in the Progression of Squamous Cell Carcinoma. Cancer Sci..

[15] Wang G., Zhang M., Cheng M., Wang X., Li K., Chen J., Chen Z., Chen S., Chen J., Xiong G. (2021). Tumor Microenvironment in Head and Neck Squamous Cell Carcinoma: Functions and Regulatory Mechanisms. Cancer Lett..

[16] Clayton S.M., Archard J.A., Wagner J., Farwell D.G., Bewley A.F., Beliveau A., Birkeland A., Rao S., Abouyared M., Belafsky P.C. (2020). Immunoregulatory Potential of Exosomes Derived from Cancer Stem Cells. Stem Cells Dev..

[17] Saikia P.J., Pathak L., Mitra S., Das B. (2023). The Emerging Role of Oral Microbiota in Oral Cancer Initiation, Progression and Stemness. Front. Immunol..

[18] Griso A.B., Acero-Riaguas L., Castelo B., Cebrián-Carretero J.L., Sastre-Perona A. (2022). Mechanisms of Cisplatin Resistance in HPV Negative Head and Neck Squamous Cell Carcinomas. Cells.

[19] Hu H., Li B., Wang J., Tan Y., Xu M., Xu W., Lu H. (2023). New Advances into Cisplatin Resistance in Head and Neck Squamous Carcinoma: Mechanisms and Therapeutic Aspects. Biomed. Pharmacother..

[20] Huang J., Li H., Yang Z., Liu R., Li Y., Hu Y., Zhao S., Gao X., Yang X., Wei J. (2024). SALL4 Promotes Cancer Stem-like Cell Phenotype and Radioresistance in Oral Squamous Cell Carcinomas via Methyltransferase-like 3-Mediated m6A Modification. Cell Death Dis..

[21] Hutchinson M.-K.N.D., Mierzwa M., D’Silva N.J. (2020). Radiation Resistance in Head and Neck Squamous Cell Carcinoma: Dire Need for an Appropriate Sensitizer. Oncogene.

[22] Ruan S., Lin M., Zhu Y., Lum L.G., Thakur A., Jin R., Shao W., Zhang Y., Hu Y., Huang S. (2020). Integrin Β4-Targeted Cancer Immunotherapies Inhibit Tumor Growth and Decrease Metastasis. Cancer Res..

[23] Rodriguez-Ramirez C., Nör J.E. (2018). P53 and Cell Fate: Sensitizing Head and Neck Cancer Stem Cells to Chemotherapy. Crit. Rev. Oncog..

[24] Peitzsch C., Nathansen J., Schniewind S.I., Schwarz F., Dubrovska A. (2019). Cancer Stem Cells in Head and Neck Squamous Cell Carcinoma: Identification, Characterization and Clinical Implications. Cancers.

[25] Fan Z., Li M., Chen X., Wang J., Liang X., Wang H., Wang Z., Cheng B., Xia J. (2017). Prognostic Value of Cancer Stem Cell Markers in Head and Neck Squamous Cell Carcinoma: A Meta-Analysis. Sci. Rep..

[26] Elkashty O.A., Ashry R., Tran S.D. (2019). Head and Neck Cancer Management and Cancer Stem Cells Implication. Saudi Dent. J..

[27] Takebe N., Miele L., Harris P.J., Jeong W., Bando H., Kahn M., Yang S.X., Ivy S.P. (2015). Targeting Notch, Hedgehog, and Wnt Pathways in Cancer Stem Cells: Clinical Update. Nat. Rev. Clin. Oncol..

[28] Shahoumi L.A. (2021). Oral Cancer Stem Cells: Therapeutic Implications and Challenges. Front. Oral Health.

[29] Kerk S.A., Finkel K.A., Pearson A.T., Warner K.A., Zhang Z., Nör F., Wagner V.P., Vargas P.A., Wicha M.S., Hurt E.M. (2017). 5T4-Targeted Therapy Ablates Cancer Stem Cells and Prevents Recurrence of Head and Neck Squamous Cell Carcinoma. Clin. Cancer Res..

[30] Sun Q., Chen X., Luo H., Meng C., Zhu D. (2023). Cancer Stem Cells of Head and Neck Squamous Cell Carcinoma; Distance towards Clinical Application; a Systematic Review of Literature. Am. J. Cancer Res..

[31] Alix-Panabières C., Pantel K. (2021). Liquid Biopsy: From Discovery to Clinical Application. Cancer Discov..

